# In situ forming ROS-scavenging hybrid hydrogel loaded with polydopamine-modified fullerene nanocomposites for promoting skin wound healing

**DOI:** 10.1186/s12951-023-01879-2

**Published:** 2023-04-13

**Authors:** Xuan Chen, Yihui Zhang, Wei Yu, Wenkai Zhang, Haozheng Tang, Wei-En Yuan

**Affiliations:** 1 Engineering Research Center of Cell & Therapeutic Antibody, Ministry of Education, School of Pharmacy, Shanghai Jiao Tong University, Shanghai, 200240 PR China; 2 Department of Bone and Joint Surgery, Department of Orthopedics, Renji Hospital, School of Medicine, Shanghai Jiao Tong University, 145 Shandong Middle Road, Shanghai, 200001 PR China

**Keywords:** Hybrid hydrogel, Fullerene, Polydopamine, Antioxidant, Wound healing

## Abstract

**Background:**

Excessive oxidative stress at the wound sites always leads to a prolonged healing and even causes chronic inflammatory wounds. Therefore, antioxidative dressings with multiple features are desired to improve wound healing performance. Herein, we fabricated a ROS-scavenging hybrid hydrogel by incorporating mussel-inspired fullerene nanocomposites (C60@PDA) into gelatin methacryloyl (GelMA) hydrogel.

**Results:**

The developed C60@PDA/GelMA hydrogel showed a sustainable free radical scavenging ability, and eliminated ROS to protect cells against external oxidative stress damage. Besides, the hydrogel presented favorable cytocompatibility, hemocompatibility, and antibacterial ability in vitro. Furthermore, in a mouse full-thickness wound defect model, the in situ forming hybrid hydrogel accelerated wound closure by 38.5% and 42.9% on day 3 and day 7 over the control. Histological results demonstrated that hybrid hydrogels effectively enhanced wound healing on re-epithelialization, collagen deposition and angiogenesis.

**Conclusion:**

Collectively, the C60@PDA/GelMA hydrogel could be a promising dressing for promoting cutaneous wound repair.

**Supplementary Information:**

The online version contains supplementary material available at 10.1186/s12951-023-01879-2.

## Introduction

Skin, as the first barrier to defense against dehydration, physical injuries and micro-organisms invasion, plays an essential role in maintaining homeostasis [1, 2]. However, skin damage would result in the loss of its protective functions, and even endanger life, especially for large skin wounds. Approximately 8.2 million people suffer from at least one type of wound and it was estimated that the global wound closure products will exceed $22 billion worldwide by 2024 [3, 4].

Various wound dressings, including films, nanofibrous membranes and hydrogels, have been developed to promote wound healing instead of traditional cotton wool and gauze [1, 5–7]. Hydrogels are considered the most promising choice among these wound dressings. Their high-water content and three-dimensional microstructure endow the wound with a moist and extracellular matrix (ECM)-like microenvironment for wound healing [8, 9]. Recently, hydrogels based on gelatin have been widely explored for wound healing. Gelatin is a natural polymer derived from collagen. Owing to the arginine-glycine-aspartic acid (RGD) peptides structure, gelatin allows cell adhesion and migration, showing potential for tissue engineering [10]. However, its poor mechanical properties and undesirable processing conditions call for suitable modification [11]. One of the most common strategies is the methylacrylylation of gelatin (GelMA). Studies have shown that combing GelMA hydrogel with functional materials could endow the scaffold with superior properties such as conductivity, anti-inflammatory and anti-bacterial [12–14].

Furthermore, it should be noted that advanced hydrogels with some unique properties would be more attractive for wound dressing, because of the dynamic and complicated healing process, including hemostasis, inflammation, proliferation and remodeling [15]. The inflammatory phase initiates from the occurrence of skin injury, causing the excessive production of reactive oxygen species (ROS) [9]. Excess levels of ROS hinder the wound’s transition from the inflammation phase to the proliferation phase, resulting in a persistent inflammatory state. Numerous antioxidative substances, such as ascorbic acid, cysteine, tannic acid and curcumin have been incorporated into hydrogel dressings for accelerating wound healing [16–19]. Due to its exceptional antioxidant capacity, fullerene called “radical sponge” has emerged as a potential functional material for skincare. It has been widely applied for whitening, anti-aging, and sunscreen cosmetics [20–22]. Besides, its other properties like anti-inflammatory, anti-viral, antibacterial and hair growth stimulating have drawn considerable attention among researchers [20, 23, 24]. Nonetheless, investigations about fullerene hydrogels for wound care have not been reported yet. It is reasonably expected that such hybrid hydrogels show great potential for cutaneous wound healing. However, pristine fullerene is almost insoluble in aqueous media, which precludes its potential biological applications. To address this issue, many strategies have been developed to improve fullerene bioavailability, including encapsulation, formation of suspension and chemical modification [25, 26]. Inspired by natural mussel adhesion chemistry, polydopamine (PDA) has been widely employed for surface functionalization owing to its high catechol and amine contents, and the PDA-coated nanomaterials also exhibited appropriate biocompatibility, tissue adhesiveness and desirable antibacterial capacity [27–30].

**Fig. 1:**
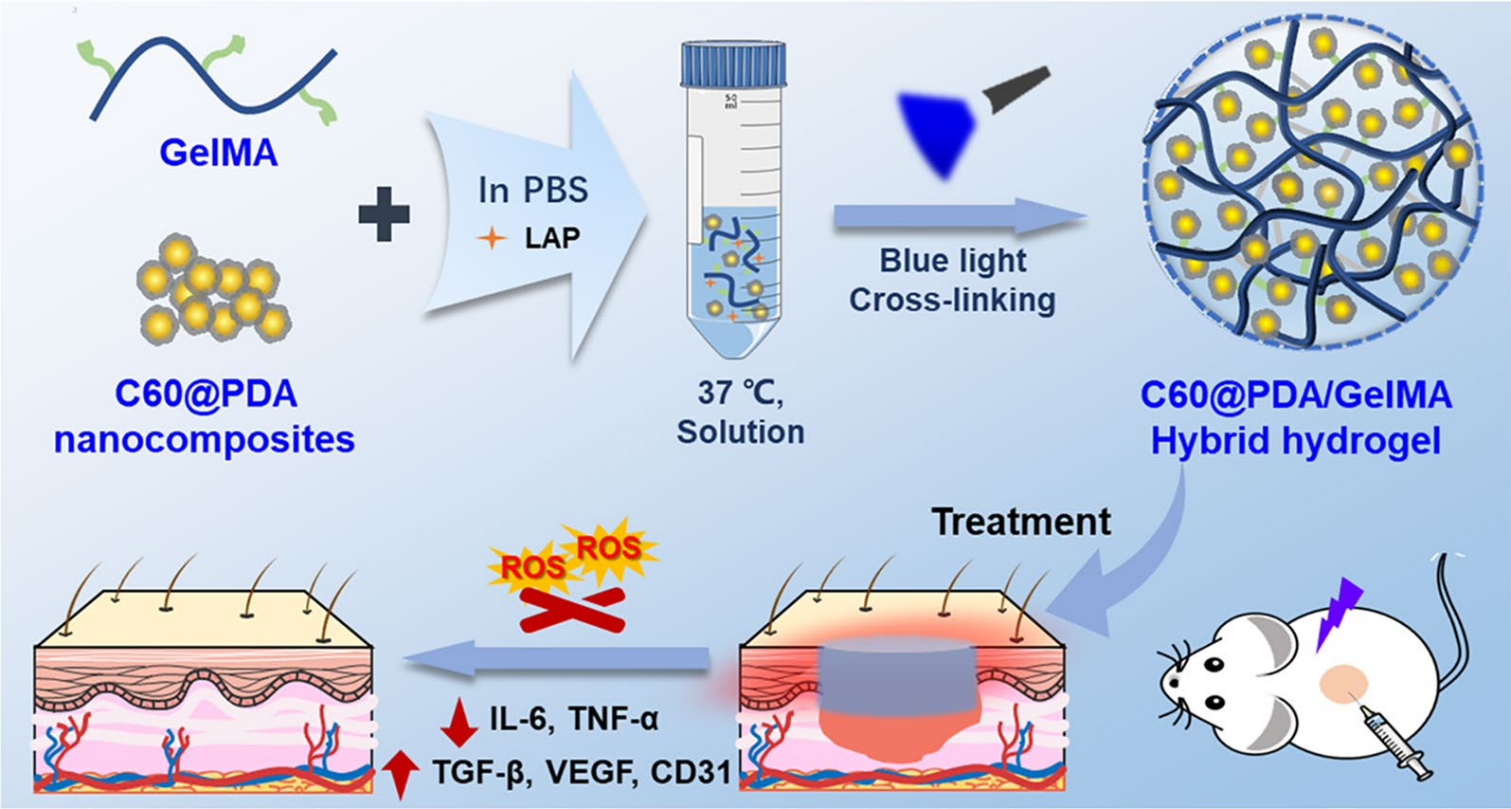
Schematic illustration of the fabrication and application of C60@PDA/GelMA hybrid hydrogel

Herein, we fabricated an in situ forming hybrid hydrogel consisting of GelMA and PDA-coated fullerene (C60@PDA) for wound repair. C60@PDA was produced via a mild and green strategy, and then it was incorporated into the GelMA to form a hybrid hydrogel in situ via photo-crosslinking (Fig. 1). The physical and chemical properties of the prepared hybrid hydrogels were systematically characterized to confirm their feasibility to provide a supportive antioxidative microenvironment for wound dressing. The biocompatibility of hydrogels was assessed by co-culturing with mouse fibroblast (L929) cells and hemolytic assay, and the antibacterial activity was evaluated by a surface antibacterial experiment. In vivo, a full-thickness wound defect model of rats was established to investigate the therapeutic effects of the hybrid hydrogel including analysis of wound closure and histological staining.

## Materials and methods

### Materials

Type-A gelatin from porcine skin (~ 300 g bloom, reagent grade), methacrylic anhydride (MA), and dopamine hydrochloride (DA∙HCl) were purchased from Sigma-Aldrich (Shanghai, China). Fullerene (C60, 99.9%) was purchased from Funano Co. Ltd. (Xiamen, China). Hydrogen peroxide (H_2_O_2_, 30%) was purchased from Lingfeng Chemical (Shanghai, China). 2-Amino-2-(hydroxymethyl)-1,3-propanediol (Tris) was from BBI Life Sciences Co. Ltd. (Shanghai, China). Photoinitiator lithium phenyl-2,4,6-trimethylbenzoylphosphinate (LAP) was purchased from EFL (Suzhou, China). 1,1-Diphenyl-2-picrylhydrazyl (DPPH) was purchased from Yuanye Bio-Technology Co. Ltd. (Shanghai, China). Ferrous sulfate (FeSO_4_) and salicylic acid were purchased from Macklin Biochemical Technology Co. Ltd. (Shanghai, China). The superoxide anion assay kit was obtained from Geruisi Bio-Technology Co. Ltd. (Suzhou, China). 2′,7′-Dichlorodihydrofluorescein diacetate (DCFH-DA) assay kit was from Solarbio (Beijing, China). Mouse fibroblast (L929) cells were ordered from the cell bank of Chinese Academy of Sciences (Shanghai, China). Phosphate Buffered Saline (PBS), Dulbecco’s Phosphate Buffered Saline (DPBS), Minimum Essential Medium α (α-MEM), fetal bovine serum (FBS) and penicillin/streptomycin solution were purchased from Gibco (USA). Cell counting kit 8 (CCK-8) was purchased from Dojindo Molecular Technologies (Japan). All the antibodies were purchased from Abcam (China).

### Synthesis of GelMA

GelMA was synthesized by reacting the amino(-NH_2_) and hydroxyl(-OH) groups of gelatin with methacryloyl as previously reported [31]. Firstly, gelatin was completely dissolved in DPBS at 50 °C with stirring to obtain a 10% (w/v) solution. Next, MA (8% v/v) was slowly added to the gelatin solution at the rate of 0.1 mL min^−1^ while stirring under a dark environment, and the reaction proceeded at 50 °C for 2 h until plentiful preheated DPBS was added to stop it. The diluted mixture was centrifuged at a speed of 5000 rpm for 10 min to remove the unreacted precipitates. Then, the solution was transferred into dialysis tubes (10 kDa) and dialyzed against deionized water at 40 °C for 5 d. Finally, the dialyzed solution was lyophilized for 3 d to obtain the GelMA foam. The chemical structure was characterized by Avance III 400 MHz ^1^H NMR spectrometer (Bruker, USA), using solvent D_2_O for gelatin and GelMA.

### Synthesis and characterization of C60@PDA nanoparticles

C60@PDA nanocomposites were produced by self-polymerization of DA∙HCl with fullerene under a weakly alkaline condition. Briefly, fullerene powder (100 mg) was bath-sonicated for 30 min to obtain a well-dispersed suspension in 200 mL of 10 mM Tris-HCl buffer (pH 8.5). Then, DA∙HCl (0.5 mg mL^−1^) was added to the suspension and allowed for continuous stirring at 60 °C for 24 h. The resulting dark solution was centrifugated and washed by deionized water and ethanol five times to obtain C60@PDA nanocomposites. Approximately 90 mg lyophilized C60@PDA nanoparticles were obtained, and the lyophilized samples were characterized by Fourier transform infrared spectrometer (FTIR, Nicolet 6700). X-ray photoelectron spectroscopy (XPS) of C60 and C60@PDA was measured by AXIS UltraDLD (Shimadzu). The morphologies of C60 and C60@PDA were observed by a transmission electron microscope (TEM, Talos L120C G2, Thermofisher). The size distribution and zeta potential of C60 and C60@PDA were examined by dynamic light scattering (DLS, Nano ZS90, Malvern Instrument Ltd.). Thermogravimetric analyses were conducted on the simultaneous thermal analyzer (SDT-Q600, TA).

### Preparation of GelMA and C60@PDA/GelMA hybrid hydrogels

GelMA foam was dissolved in PBS (5 wt%) at 40 °C for 1 h, and 0.25 wt% photoinitiator LAP was added to form the GH prepolymer solution. Prepared C60@PDA dispersion was mixed with GelMA solution to prepare prepolymer C60@PDA/GelMA solutions with a concentration of 0.5 mg mL^−1^. Then, the hydrogels were prepared under UV exposure (405 nm) in the cylindrical molds or 24-well plates for 30 s, denoted as GH and CPGH in the figures. To observe the morphology of hydrogels, they were lyophilized for 3 d and coated with a layer of gold. The morphology of hydrogels was observed by scanning electron microscopy (SEM, Sirion 200/IAC, SJTU) with an accelerating voltage of 5 kV.

### Swelling behavior and degradation assay

The lyophilized hydrogel samples (Φ 5 mm × 4 mm) were immersed into PBS (pH 7.4) at 37 °C to study the swelling kinetics. At predetermined time points, the hydrogels were picked out and the weight (W_t_) was recorded after being wiped by filter papers. The weight of the initially dried hydrogels was recorded as W_0_. The swelling ratio of hydrogels was calculated as the following formula:

1 $$Swelling\;ratio \left( \% \right) = \left( {W_{t} - W_{0} } \right)/W_{0} \times 100\% .$$

To evaluate the degradation of hybrid hydrogels in vitro, the hydrogel samples (Φ 8 mm × 10 mm) were weighed (W_0_′) and then immersed in 2 mL PBS containing 1 U mL^−1^ of collagenase at 37 °C. The samples were collected at certain time points and then weighed (W_t_′) after lyophilization. The rate of degradation was estimated by remaining weight using the following formula:

2 $$Remaining\;weight \left( \% \right) = W_{t}^{^{\prime}} /W_{0}^{^{\prime}} \times 100\% .$$

### Rheological measurements

The rheological characteristics of hydrogels were analyzed by using a rheometer (ARG-2, TA, USA). GH and CPGH hydrogels were placed on a 40 mm parallel plate. The frequency sweep measurements were performed at 25 °C with a fixed strain of 0.5%. To examine the shear viscosity, the shear rate was increased from 0.1 to 100 s^−1^ at 25 °C.

### Mechanical test

Cylindrical hydrogels (Φ 8 mm × 10 mm) were placed on the platform of the dynamic thermomechanical tester (Q850, TA, USA), and the clamps were adjusted to keep in contact with the hydrogel without force. Then, the samples were compressed at a rate of 0.5 mm min^−1^ at room temperature until fracture to obtain compressive stress–strain curves.

### ROS scavenging activity tests

The antioxidant capacity of C60@PDA nanoparticles and hydrogels was firstly evaluated by DPPH free radicals scavenging assay. Briefly, prepared nanoparticles were dispersed in methanol with concentrations varying from 7.8125 to 1000 μg mL^−1^, and 200 μM DPPH/methanol was mixed with the dispersions. After incubation at room temperature in a dark environment for 30 min, the remaining mixture was centrifuged and DPPH free radical content was measured via testing absorbance at 517 nm of the supernatant, which was recorded as A_sample_. For the hydrogel samples, the cylindrical GH and CPGH hydrogels (Φ 5 mm × 4 mm) were immersed into 200 μM DPPH/methanol and were incubated in the dark for a predetermined time. The control group altered the samples with methanol, and the absorbance was recorded as A_control_. The DPPH scavenging percentage of C60@PDA and hydrogels was calculated according to the following formula:

3 $$DPPH\;scavenging\;ability \left( \% \right) = \left( {1 - A_{Sample} /A_{Control} } \right) \times 100\% .$$

The hydroxyl radicals (·OH) and superoxide anions (O_2_^**.**−^) scavenging effects of the hydrogels were subsequently evaluated. The ·OH scavenging ability was assessed via Fenton reaction. Briefly, 10 mM FeSO_4_ and 10 mM H_2_O_2_ were added to the hydrogel samples (Φ 5 mm × 4 mm). After 10 min of incubation, 10 mM salicylic acid-ethanol was added, and the mixture was incubated in the dark for 30 min. Then, the absorbance of the solution at 510 nm was measured to calculate ·OH scavenging capacity. The O_2_^**.**−^ scavenging assay was performed according to the kit instructions via pyrogallol autoxidation. The hydrogel samples (Φ 5 mm × 4 mm) were incubated at room temperature with the reagents, and the absorbance of the solution at 320 nm was measured to calculate O_2_^**.**−^ scavenging ability.

### In vitro biocompatibility evaluation

L929 cells were used to assess the biocompatibility of nanoparticles and hydrogels. To evaluate the biosafety of C60@PDA nanoparticles in vitro, L929 cells were co-cultured with dispersion solution (0.0625–2 mg mL^−1^) for 4 h and 24 h respectively, and the cytotoxicity was analyzed by CCK-8 assay by reading the absorbance at 450 nm and 630 nm. The optimal concentration was selected according to the cell viability. Furthermore, the biocompatibility of hydrogels was evaluated by performing a direct contact between hydrogels and L929 cells. In brief, 200 μL prepolymer solution was pipetted into a 24-well plate and irradiated under UV light to form hydrogels followed by incubating with complete medium for 4 h. L929 cells were seeded onto the surface of the hydrogels at the density of 10^5^ cells/well and cultured for 12 h, 24 h and 48 h respectively. Then, the CCK-8 assay was conducted and 100 μL of supernatants were transferred into a 96-well plate and read at 450/630 nm using a microplate reader (Varioskan Flash, Thermo).

### In vitro hemocompatibility assay

The hemocompatibility of the hybrid hydrogels was assessed by the red blood cells of mouse. In brief, the blood of mouse was centrifuged at 1000 rpm for 10 min, and the red blood cells were washed with normal saline 3 times. Then it was diluted with saline to the concentration of 5% (v/v). 100 μL hydrogels were prepared in a syringe tube, and the samples were put into EP tubes with 500 μL mouse red blood cells suspension. After 1 h incubation at 37 °C, the hydrogels were taken out followed by 100 μL saline adding. Then the supernatant obtained from suspension centrifugation was tested by reading the absorbance at 540 nm. 0.1% Triton X-100 was used as the positive control and saline as the negative control. The hemolysis ratio was calculated according to the following formula:

4 $$Hemolysis\;ratio \left( \% \right) = \left( {A_{H} - A_{S} - A_{0} } \right)/\left( {A_{100} - A_{0} } \right) \times 100\% ,$$

where A_H_ was the absorbance value of the hydrogel group; A_S_ was the absorbance value of the hydrogel in saline; A_100_ was the absorbance value of the positive control Triton X-100; A_0_ was the absorbance value of saline.

### Intracellular ROS measurement

The ROS scavenging capacity of hydrogels was further studied by fluorescence staining. The extracted solutions of GH and CPGH were collected after immersing hydrogels in the medium for 24 h. L929 cells were seeded in the 6-well plate and cultured until they reached about 70% confluency. The cells were incubated with the extracted solution of GH and CPGH for 24 h and subsequently treated with 50 mM H_2_O_2_ for 30 min. The negative group was treated with fresh medium. Then, the cells were stained with DCFH-DA (10 μmol L^−1^) for 30 min and washed with PBS three times. The stained cells were observed and imaged using an inverted confocal microscope (Leica TCS SP8).

### Antibacterial activity assessment

The antibacterial activities of hybrid hydrogels were measured against *Escherichia coli* (*E. coli)* and *Staphylococcus aureus* (*S. aureus*), which are the model bacteria of gram-negative and gram-positive bacteria. Briefly, 200 μL of sterilized prepolymer solutions were placed into a 24-well plate and they were completely gelled by UV irradiation. 1 mL of bacterial suspension (10^6^ CFU mL^−1^) in Luria–Bertani (LB) broth medium was added onto the surface of hydrogels and incubated for 12 h at 37 °C. Afterward, PBS was added to resuspend the bacterial survivor followed by a series of dilutions. Then, 100 μL of bacterial suspension was spread evenly on LB agar plates. The number of colonies was counted by ImageJ software after incubation for another 14 h. The results were expressed as bacterial viability (%), which was calculated by the following equation:

5 $$Bacterial\;viability \left( \% \right) = \left( {colony\;count\;on\;hydrogels/ control} \right) \times 100\% .$$

### In vivo wound healing study

A mouse full-thickness wound defect model was established to evaluate the wound healing efficacy in vivo. Female BALB/c mice (~ 20 g) were randomly divided into four groups including PBS, GH and CPGH groups (12 mice for each group). The dorsal hair was removed with depilatory cream after anesthesia, and a full-thickness wound defect with a diameter of 10 mm was made on the mice. The prepolymer solution (60 μL) was introduced to cover the wound site with 30 s UV irradiation, while the control group was treated with PBS (60 μL). The wound sites were photographed on days 0, 3, 7, 11 and 14, and the wound areas were determined by using ImageJ software. Relative wound area was calculated as the following formula:

6 $$Relative \;wound\;area \left( \% \right) = A_{t} /A_{0} \times 100\% .$$

where A_t_ and A_0_ were the wound area sizes on day t and day 0, respectively. During the experiment process, the weight of mice was recorded at predetermined time intervals. After 3, 7, 14 and 21 days of treatment, the mice were sacrificed. The surrounding wound tissues and main organs were collected and fixed in 4% paraformaldehyde for 24 h. All animal experimental procedures were conducted according to the Guide for the Care and Use of Laboratory Animals published by the National Institutes of Health (eighth Edition, 2011), and the animal operation processes were approved by the Institutional Animal Care and Use Committee (IACUC) of Shanghai Jiao Tong University (SJTU, No. A2021096-1).

### Histological and immunohistochemical assessment

The harvested tissues were embedded into the paraffin and cross-sectioned into 5 μm-thick slices for staining. Hematoxylin and eosin (H&E) staining and Masson’s trichrome staining were performed under standard protocols to evaluate epidermal regeneration and collagen deposition in the wound site. The dermal collagen deposition was determined as collagen volume fraction (%). Besides, to explore inflammation and blood vessel regeneration, the immunohistochemistry staining with IL-6, TNF-α, VEGF, TGF-β and CD 31 was carried out according to the manufacturer’s instructions. Histological images of the stained slices were captured by optical microscope (Olympus BX53, Japan).

### Statistical analysis

All the results were expressed as means ± standard deviation (SD). Data analysis for multiple comparisons was carried out by the one-way analysis of variance (ANOVA) with GraphPad Prism software (version 8.0). p-value < 0.05 was considered as significant level (*P < 0.05, **P < 0.01, ***P < 0.001 and ****P < 0.0001).

## Results and discussion

### Synthesis and characterization of GelMA and C60@PDA

**Fig. 2:**
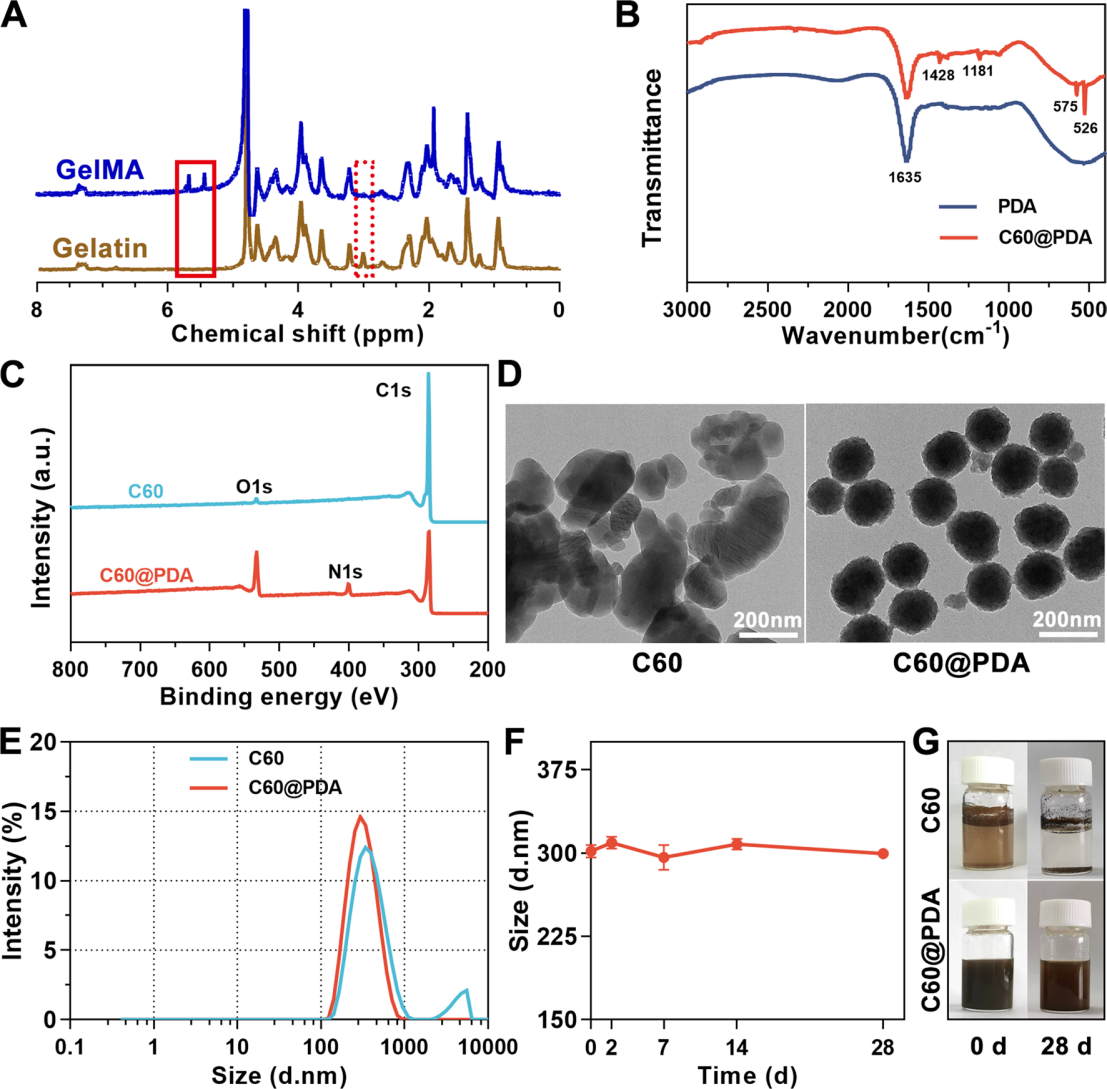
Characterizations of C60@PDA nanocomposites. **A** ^1^H NMR spectra of gelatin and GelMA. **B** FTIR spectra of PDA and C60@PDA nanocomposites. **C** XPS survey spectra of C60 and C60@PDA. **D** TEM images of C60 and C60@PDA nanoparticles. **E** Size distribution of C60 and C60@PDA nanoparticles. **F** Size change of C60@PDA nanoparticles at different times. **G** Stabilization assay of the C60 and C60@PDA nanocomposites solution: the newly prepared sample (0 d) and the sample after standing for 4 weeks (28 d)

GelMA was synthesized as the hydrogel matrix by the functionalization of gelatin, and the structure was identified by ^1^H NMR spectrometry (Fig. 2A). Two typical peaks at 5.43 ppm and 5.66 ppm were observed on the ^1^H NMR spectra of GelMA, corresponding to vinyl groups of methacrylic anhydride, while peak at 2.9 ppm was attributed to the decrease in lysine methylene of gelatin [32, 33]. The methacrylated degree was about 34%, determined by peak areas. GelMA-based hydrogels were photopolymerized in situ to accommodate the irregular wound defects, which have been given priority to the pre-fabricated scaffolds [34]. To endow the hydrogel wound dressing with ROS-scavenging function, we prepared C60@PDA nanocomposites by dopamine self-polymerization on the surface of fullerene in a weakly alkaline Tris-HCl buffer, and incorporated them into GelMA hydrogel.

The physical characteristics of C60@PDA nanocomposites were characterized. As shown in Fig. 2B, FTIR wave signals at 1428 cm^−1^, 1181 cm^−1^, 575 cm^−1^ and 526 cm^−1^ were specific signals from fullerene owing to the intramolecular vibration of C60 skeleton structure [29, 35]. XPS spectra showed a new N1s signal at 400.0 eV and an enhanced signal at 533.0 eV in C60@PDA (Fig. 2C), which were attributed to the amino groups and -OH groups on dopamine [36]. According to the XPS survey, the atomic ratio of nitrogen elements in the C60@PDA was approximately 4.83%. We also observed a weak oxygen signal in C60, probably due to the slight oxidation of fullerene. The thermostability of prepared samples was studied by thermogravimetric analysis (Additional file 1: Fig. S1). C60 performed excellent thermal stability, but significant decomposition was observed after PDA coating, mainly due to the combustion of the coated PDA. These results demonstrated that PDA was successfully modified onto the fullerene by self-polymerization.

TEM images (Fig. 2D) showed that C60@PDA exhibited a uniform spherical structure with a rough surface, while C60 nanosheets agglomerated easily. The hydrodynamic radius of the newly prepared C60@PDA nanoparticles was 301.6 nm (Fig. 2E), and the average size remained constant during the 4 weeks of placement (Fig. 2F), which was in line with the digital images of the C60@PDA water solution (Fig. 2G). In addition, C60@PDA nanocomposites had an average negative zeta potential of − 52.9 mV, suggesting the formation of a stable dispersion system (Additional file 1: Fig. S3). Accordingly, the PDA coating on the C60 could increase the dispersion stability of C60 in water, and improve the feasibility of fullerene materials for wound healing.

### Fabrication and characterization of hybrid hydrogels

**Fig. 3:**
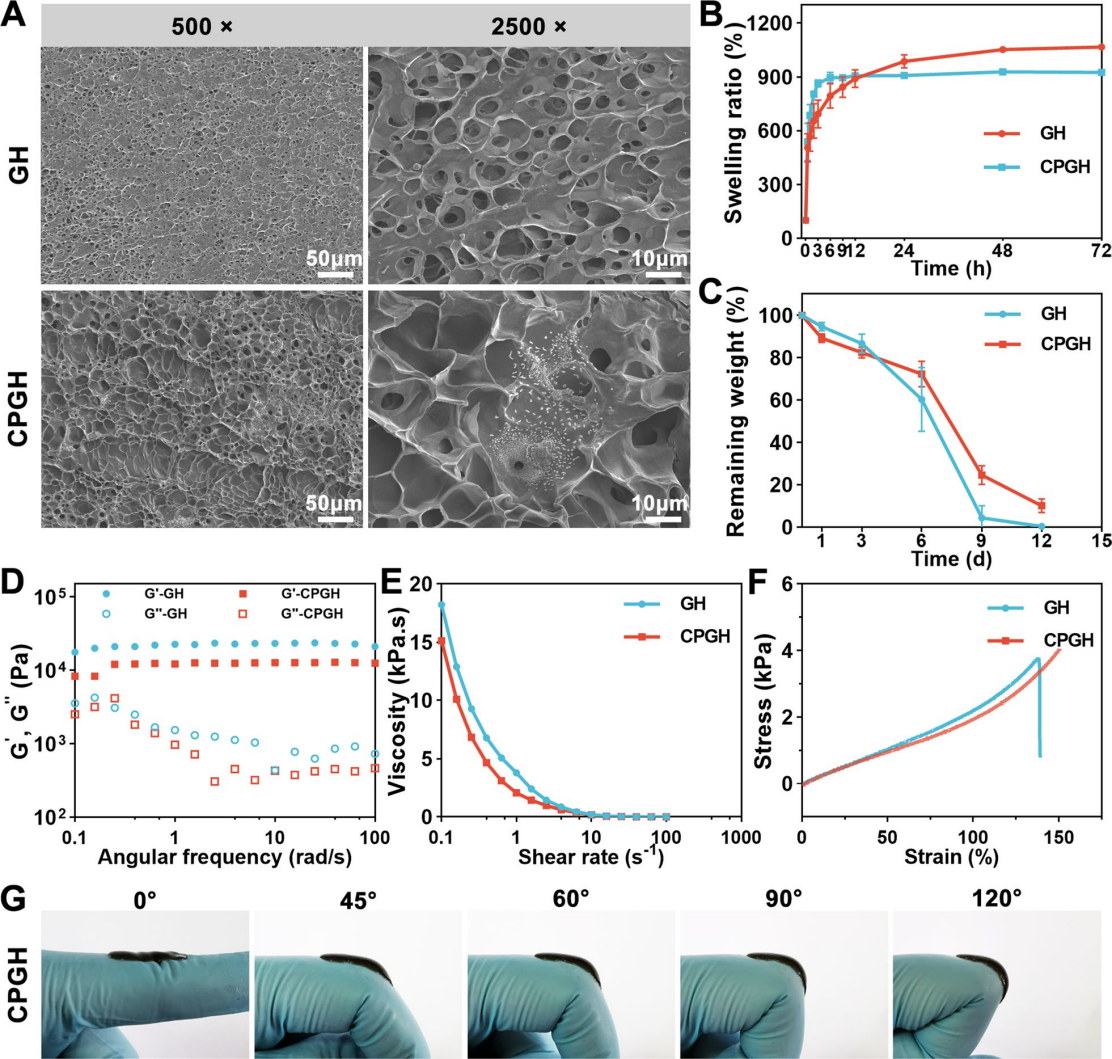
Characterizations of the hydrogels. **A** SEM images of GH and CPGH hydrogels. **B** Swelling ratio of the hydrogels in PBS (n = 3). **C** Degradation profiles of hydrogels in PBS containing 1 U mL^−1^ of collagenase at 37 °C (n = 4). **D** Rheological property of the hydrogels: frequency spectra of storage modulus (G′) and loss modulus (G″) of the hydrogel at a fixing strain of 0.5%. **E** Shear-thinning behavior of the hydrogels. **F** Compressive stress–strain curves of GH and CPGH hydrogels. **G** Photographs of the CPGH hydrogels that were applied on the human finger wrist during exercise

The hybrid hydrogels (CPGH) were fabricated by photopolymerization of GelMA solution with C60@PDA nanocomposites (Fig. 1). GelMA hydrogel (GH) was prepared and used as the control. SEM images showed that both the samples exhibited porous and sponge-like microstructures (Fig. 3A), facilitating nutrient transport and cell proliferation [37, 38]. Particularly, we observed an increase in the pore size of the hybrid hydrogel when C60@PDA nanocomposites were incorporated into the hydrogel. This indicated that C60@PDA nanocomposites might disturb the crosslinking process via intramolecular interactions with GelMA [14, 39].

To prevent wound infection, hydrogel wound dressings should absorb tissue exudates timely [8, 40]. Thus, we investigated the swelling behavior of the hydrogels** (**Fig. 3B). Compared with GH, the CPGH hydrogel swelled rapidly within the first 1 h, and reached an equilibrium state (896.2%) after 6 h, lower than GH (1052.3%). Previous studies have proved that PDA affected the interaction between the water molecules and the hydrogel crosslinking chains, leading to a decrease in the water adsorption capacity of the hybrid hydrogel [39, 41]. The in vitro degradation property of hydrogels was evaluated in PBS with collagenase. As shown in Fig. 3C, GH hydrogel almost completely degraded on day 9, while CPGH degraded just over 75%, exhibiting a lower degradation rate, which could prolong the serving time as a wound dressing.

A suitable mechanical strength is another requirement for an ideal hydrogel dressing. The rheological tests showed the storage modulus (G') value of the hydrogels maintained when the strain was fixed at 0.5%, and G′ exceeded G″, suggesting the formation of elastic and stable hydrogels (Fig. 3D). Moreover, the storage modulus was over 10^4^ Pa, indicating that GelMA-based hydrogels could maintain their integrity to provide sufficient protection for the wound [42]. The GH and CPGH also exhibited a shear thinning behavior (Fig. 3E), allowing the hybrid hydrogel to flow as a viscous solution under an applied shear force and mimic the ECM. The compressive stress–strain curves were displayed in Fig. 3F. GH exhibited a low compressive strength of 3.7 kPa and a compressive break strain of 135%. In contrast, the CPGH remained intact and elastic when reaching the maximum strain of 150% owing to non-covalent interactions in the hydrogel network, which could comfort unexpected deformations of the wound [14]. Additionally, we employed the CPGH on the human wrist to identify its feasibility as a wound dressing for dynamic wounds (Fig. 3G). The CPGH could firmly adhere to the finger joint and adapt to different angles, suggesting good stretchability.

### Antioxidant capacity and in vitro biocompatibility evaluation

**Fig. 4:**
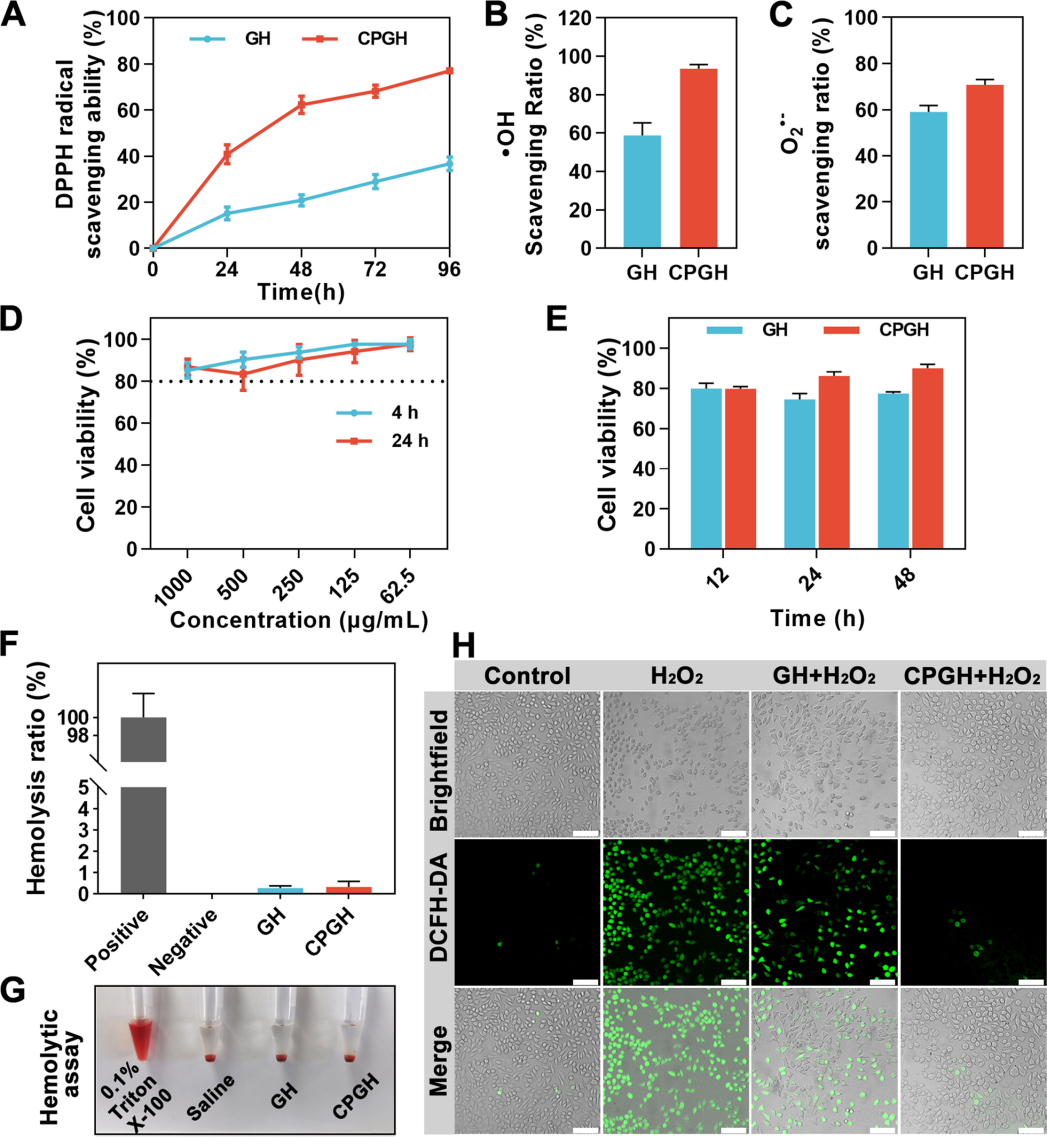
Antioxidant capacity and in vitro biocompatibility tests of hydrogels. **A** DPPH radical scavenging percentage of GH and CPGH. Free radical scavenging ratio of GH and CPGH against (**B**) ·OH and (**C**) O_2_^**.**−^. **D** Cell viability of L929 cells after incubating 4 h and 24 h with C60@PDA of different concentrations. **E** Cell viability of L929 cells after contacting with GH and CPGH hydrogels for 12 h, 24 h and 48 h. **F** In vitro hemolysis ratios of hydrogels; the positive group: Triton X-100; the negative group: saline solution. **G** The hemolytic assay of hydrogels: Photographs of supernatants after being contacted with Triton X-100, saline, GH and CPGH. **H** DCFH-DA assay for detecting ROS levels: DCF fluorescence staining images of L929 cells treated with hydrogel extracts after pretreatment with H_2_O_2_, scale bar: 100 μm. (n = 4, mean ± S.D.)

Overexpression of ROS hinders the wound healing process, and antioxidant hydrogel dressings have attracted more attention due to their ROS scavenging effects [9, 43]. The antioxidant activity of C60@PDA nanocomposites was firstly evaluated by DPPH free radical scavenging assay. C60@PDA scavenged nearly 100% DPPH radical even at a low concentration of 31.25 μg mL^−1^, and the solution color changed from purple to light yellow (Additional file 1: Fig. S2). Then, we evaluated the ROS scavenging property of the hydrogels. With the incorporation of C60@PDA, the hybrid hydrogels showed greater DPPH radical scavenging ability than GH (Fig. 4A). The CPGH hydrogel could scavenge DPPH free radicals gradually and reached 77% until 96 h, which was beneficial for the sustainable antioxidant activity. Hydroxyl radicals and superoxide anions are typical oxygen free radicals, which would damage the redox balance. As shown in Fig. 4B, C, the ·OH and O_2_^**.**−^ scavenging efficiency of the CPGH hydrogel could reach 93% and 70%, respectively. The CPGH group showed better performance than GH group, demonstrating that the hybrid hydrogel could scavenge oxygen free radicals well. The pure GelMA hydrogel could eliminate nearly 60% of the ·OH and O_2_^**.**−^, probably due to the electron-donating groups such as hydroxyl and amine groups [10, 44]. These results indicated that C60@PDA/GelMA hydrogel could scavenge multiple types of ROS. The free radical scavenging ability of C60@PDA was ascribed to the redox chemistry of catechol, and the delocalized π double bond system that can attach various free radicals [45–47]. Besides, to optimize the concentration of nanocomposites incorporated into the hydrogel, L929 cells were co-cultured with various concentrations of C60@PDA for 4 h and 24 h. As shown in Fig. 4D, over 80% of the L929 cells remained viable with the concentration of C60@PDA increasing to 1000 μg mL^−1^. Based on the DPPH scavenging ability and cell viability, we selected 0.5 mg mL^−1^ as an appropriate concentration to fabricate hybrid hydrogels, fulfilling both antioxidative and biocompatible properties.

In addition, the direct contact cytotoxicity of GH and CPGH hydrogels on L929 cells was assessed by CCK-8 assay. As shown in Fig. 4E, the hybrid hydrogels exhibited good cytocompatibility, and we found that the cell viability of the CPGH was a little higher than that of the GH. This might be attributed to the enhanced cell adhesion owing to the interaction between catechol hydroxyl groups and the cell membranes, which promoted cell spreading, proliferation and migration [38, 48, 49]. We also evaluated the in vitro hemocompatibility of the hybrid hydrogels. The groups treated with Triton X-100 (hemolysis ratio: 100%) and the saline solution (hemolysis ratio: 0%) were the positive and the negative control. As shown in Fig. 4F, G, the supernatant of both the GH and CPGH group was clear and transparent, similar to the negative group. The quantitative analysis of the hemolysis ratio demonstrated that the hydrogels had a low hemolysis ratio < 2%, confirming the good hemocompatibility of the hydrogels.

Oxidative stress caused by excessive ROS at the wound sites damages the surrounding cells and prolongs healing [50]. To evaluate the intracellular ROS scavenging activity of the prepared hydrogels, we conducted the DCFH-DA staining assay. DCFH-DA could penetrate cells and be oxidized by intracellular ROS to generate fluorescent DCF, thus it was used as a detective probe for intracellular ROS levels. The L929 cells were treated with H_2_O_2_ to generate ROS, and then incubated with the extractions of hydrogels. As shown in Fig. 4H, the group treated only with H_2_O_2_ showed the highest green fluorescence intensity, indicating an increased intracellular ROS level. While little fluorescence was observed in the CPGH-treated group, and the quantitative result of fluorescence intensity was similar to the control group without H_2_O_2_ treatment (Additional file 1: Fig. S4). This suggested that the prepared C60@PDA/GelMA hybrid hydrogel could alleviate the oxidative stress in wounds, showing great potential for wound dressing application.

### In vitro antibacterial capacity

**Fig. 5:**
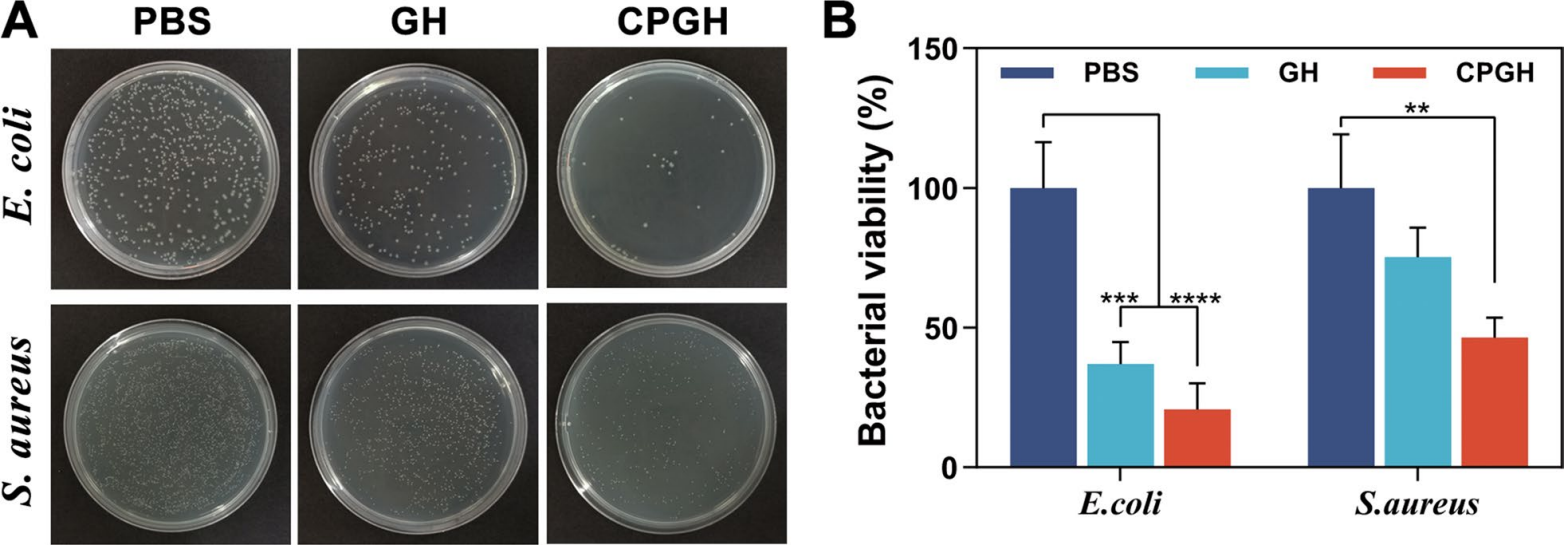
In vitro antibacterial activity of the hydrogels. **A** Images of bacteria colonies of *E. coli* and *S. aureus* on agar plates after contacting with the hydrogels for 12 h. **B** Surface bacterial viability of hydrogels for *E. coli* and *S. aureus* by colony counting. (n = 3, mean ± S.D., **P < 0.01, ***P < 0.001 and ****P < 0.0001)

Microbial infection during the healing process causes delayed healing and the formation of non-functional scars, especially for chronic wounds [51]. Thus, considerable efforts have been made on developing antibacterial hydrogel wound dressings to alter the traditional antibiotics [27, 52, 53]. To assess the antibacterial capacity of the hybrid hydrogels, we conducted a plate colony counting test. As expected, the hydrogel-treated groups displayed a remarkable decline in the bacterial colonies compared to the PBS group (Fig. 5A). Quantitative analysis of bacterial viability was shown in Fig. 5B. CPGH performed significant improved antibacterial activity in comparison with the control group for both *E. coli* and *S. aureus*. Previous studies have proved that fullerene possesses an antibacterial effect due to the bacterial membrane damage caused by electrostatic interaction with proteins [52]. Besides, the PDA coating could also play a contact-active antibacterial effect owing to the catechol groups and protic amine groups [51]. Therefore, the antibacterial capacity of CPGH could be ascribed to the combined effect of fullerene and PDA, and the exact mechanisms deserved more investigation.

### In vivo wound healing

**Fig. 6:**
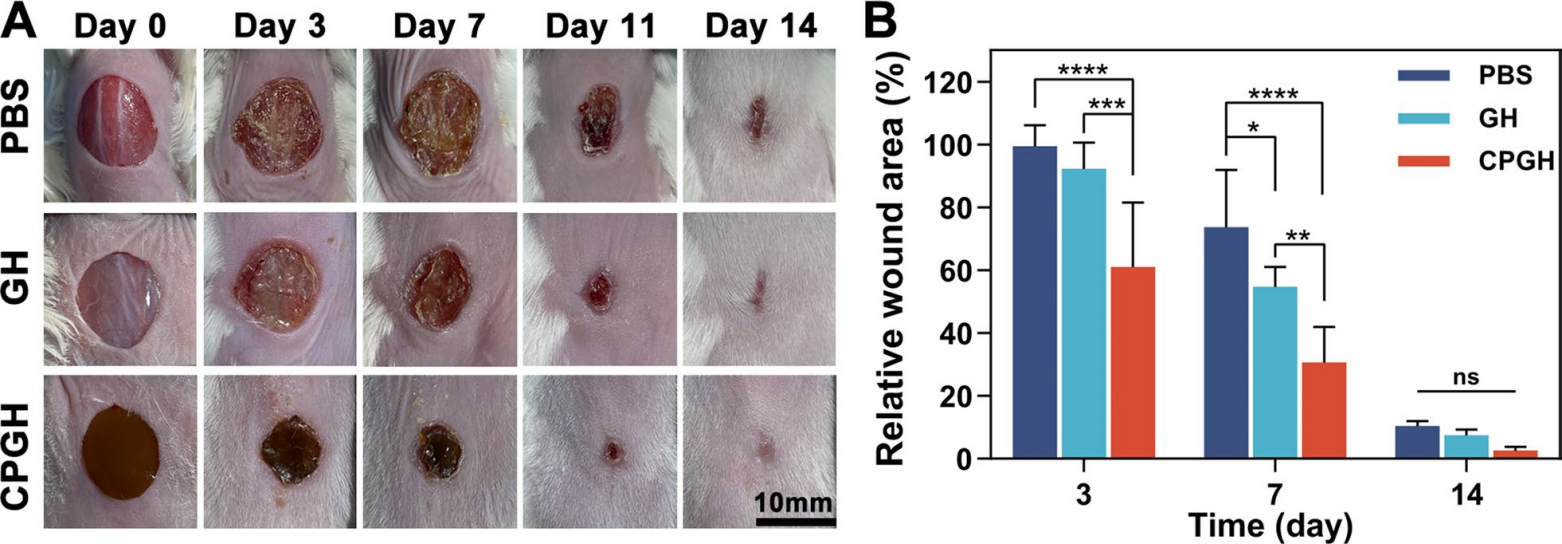
Wound closure analysis in vivo. **A** Photographs of wounds on days 0, 3, 7, 11, and 14 after treatment. **B** Wound closure analysis from the existing wound area on days 3, 7, and 14 after treatment. (n = 3, *P < 0.05, **P < 0.01, ***P < 0.001 and ****P < 0.0001)

The wound healing performance of the hybrid C60@PDA/GelMA hydrogels was evaluated in the mouse skin full-thickness defect model. The hydrogel prepolymer solution was injected into the wound site, followed by UV irradiation to form hydrogel dressings in situ. Wounds treated with PBS were used as the control. We monitored the wound healing process by taking photographs at predetermined time points (Fig. 6A). It was obvious that all the wound defects decreased over time, and the hydrogel-treated groups exhibited a higher healing efficiency than the PBS group during the wound healing process. Moreover, the percentage of the remained wound areas compared to the initial wounds was used to assess the wound closure speed (Fig. 6B). On day 3, the existing wound area of the CPGH group (60.9%) was significantly smaller than GH (92.3%) and PBS (99.4%) group, indicating CPGH was superior on accelerating wound healing. A similar trend was observed on day 7. The wound area of the CPGH group reduced to 30.7%, significantly faster than GH (54.7%) group, and the PBS group showed the slowest wound closure rate. Finally, there was no typical remaining wound area in the hydrogel-treated group on day 14, while the PBS group remained a blood clot scab. In summary, CPGH had a better wound healing performance, which might be explained that C60@PDA improved ROS-scavenging capacity to promote skin repair.

### Histological analysis

**Fig. 7:**
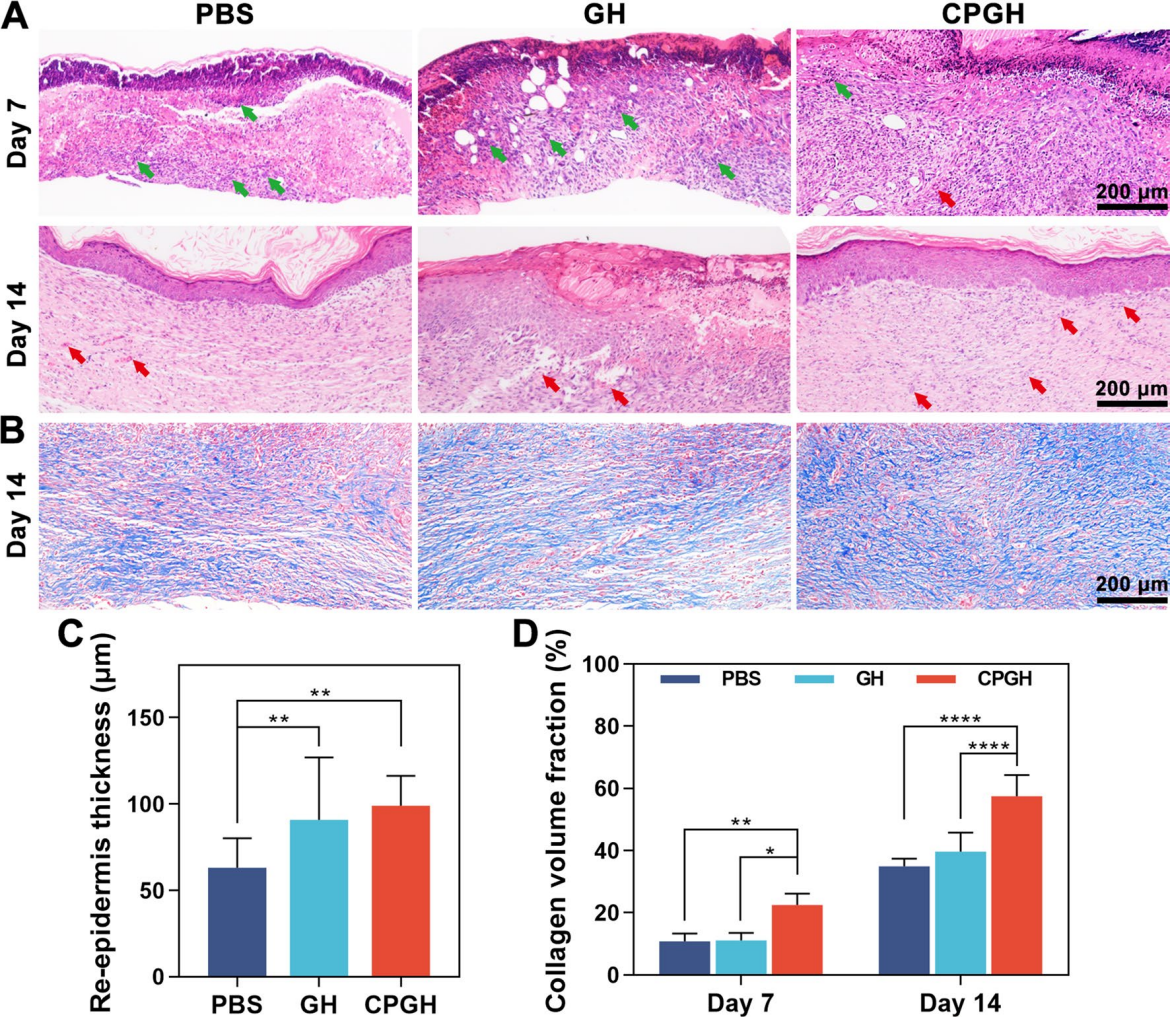
Histological analysis of the wound healing effect. **A** H&E staining of the skin wound sections by ×10 magnification on day 7 and day 14 after treatment. Red arrow: blood vessels; green arrow: inflammatory cells. **B** Masson’s trichrome staining of the wound tissues on day 14 post-treatment. **C** Quantification of re-epidermis thickness on day 14 post-treatment. **D** Quantitative analysis of collagen content (%) after 7 and 14 days of treatment. *P < 0.05, **P < 0.01, ***P < 0.001 and ****P < 0.0001

The wound healing performance of different hydrogels in vivo was further investigated by histological staining. Hematoxylin and eosin (H&E) staining was conducted on the regenerated skin after 7 and 14 days of treatment. The histopathological analysis of main organs on day 7 and day 14 post-operation proved that hydrogels were non-toxic in vivo (Additional file 1: Figs. S5, S6). As shown in Fig. 7A, all the groups showed some inflammatory cells and regenerated granulation tissues on day 7. Compared with the PBS and GH groups, hybrid hydrogels showed fewer inflammatory cells and more fibroblast infiltration. Meanwhile, some neo-vascularization was observed in the CPGH groups, which was beneficial for nutrition diffusion and wound contraction. On day 14, more blood vessels were formed in the wounds treated with hybrid hydrogels. And relatively thicker epidermis was witnessed in the hydrogel-treated groups, indicating a better wound healing effect. Since re-epithelialization has been considered to indicate the healing effect, we analyzed the thickness of the regenerated epidermis (Fig. 7C). After 14 days of treatment, the regenerated epidermis thickness of the PBS group was 63.1 μm, which was significantly lower than that of the GH (90.6 μm) and CPGH (98.9 μm) groups. On the other hand, some previous studies have shown that hypertrophy of the epidermis might cause scar formation during the later healing period [54, 55], thus the regenerated epidermis was further observed on day 21 post-operation (Additional file 1: Fig. S7). The regenerated skin in the CPGH group exhibited well-proliferated fibroblast, mature epidermis and the smallest scar area, indicating better performance on skin repair. Interestingly, a few regenerated hair follicles in the granulation tissue were observed in the CPGH groups, mainly due to the antioxidative activity of fullerene as previously reported [24]. Subsequently, Masson’s trichrome staining was performed to evaluate the formation of collagen fibers on day 14 after treatment (Fig. 7B). The collagen fibers of regenerated tissues tended to be denser and better arranged in the groups treated with the hybrid hydrogels. We then analyzed the collagen volume fractions of different groups on day 7 and day 14 after treatment (Fig. 7D, Additional file 1: Fig. S8). The collagen contents kept increasing during the 14 days of treatment. On day 14, the collagen content of CPGH (57.5%) remained higher than that of GH (39.7%) and PBS (34.9%) groups, suggesting better tissue repair performance [55].

### Anti-inflammatory and angiogenesis effect

**Fig. 8:**
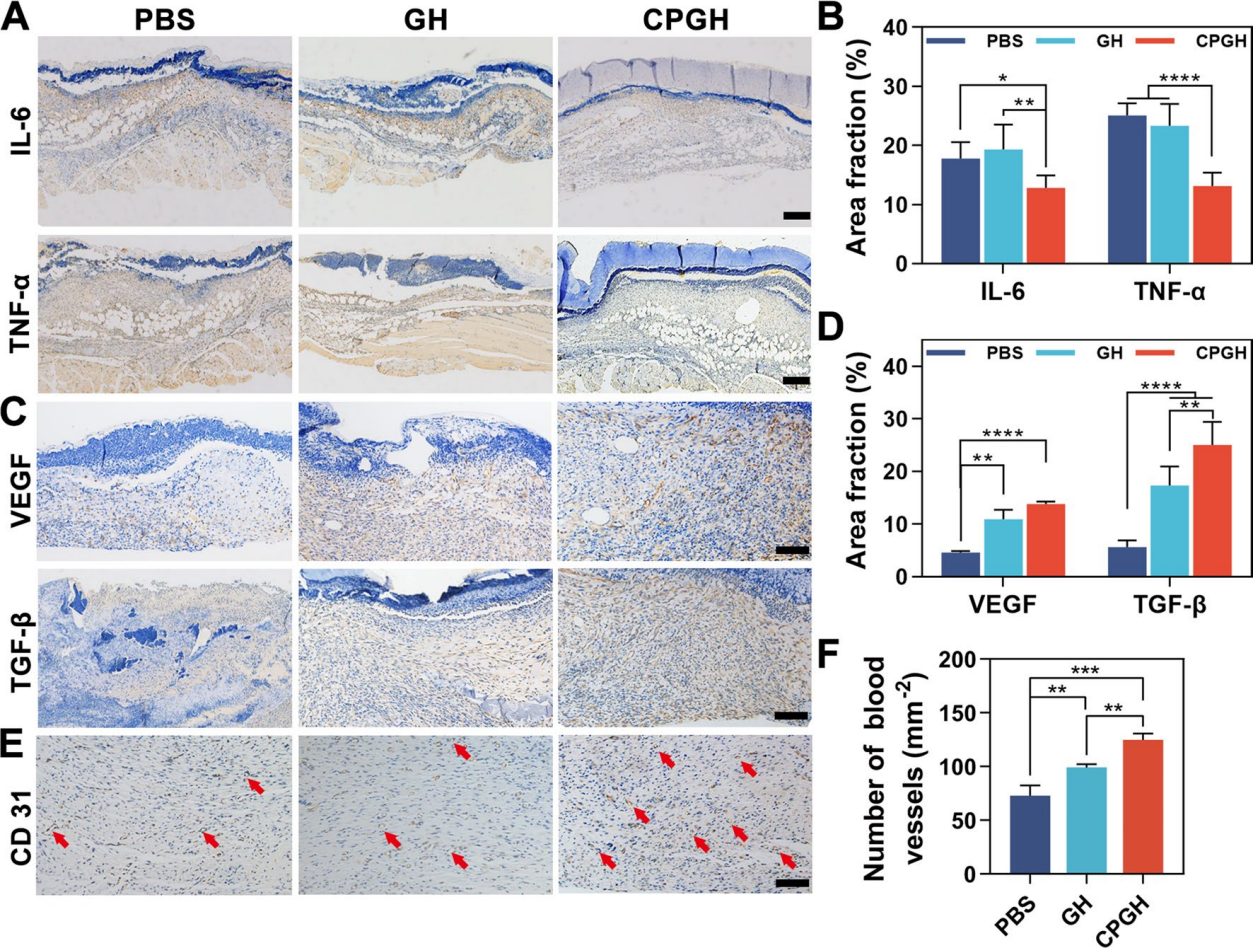
Anti-inflammatory and angiogenesis effect analysis of the hybrid hydrogels. Immunohistochemistry staining images of **A** IL-6, TNF-α on day 3 (scale bar: 200 μm), **C** VEGF, TGF-β on day 7 (scale bar: 100 μm) and **E** CD 31 at the wound sites on day 14 post-treatment (scale bar: 100 μm). Red arrows represent blood vessels. Quantitative analysis of the expression level of **B** IL-6, TNF-α and **D** VEGF, TGF-β. **F** Quantitative results of blood vessels on day 14 post-operation. *P < 0.05, **P < 0.01, ***P < 0.001 and ****P < 0.0001

In the inflammatory phase of the wounds, excessive ROS or microbial infection will cause a sustained inflammatory response, impeding the transition to the proliferative phase [9, 56]. To better demonstrate the wound healing progress, we measured the expression of main cytokines and growth factors including IL-6, TNF-α, VEGF and TGF-β. IL-6 and TNF-α were indicators of proinflammatory cytokines, which could be used to evaluate the inflammation regulation effect [57, 58]. As shown in Fig. 8A, B, the CPGH group had lower expression of IL-6 and TNF-α than both of the GH and PBS groups after 3 days of treatments, indicating a shorter inflammatory response than a normal process and further confirming the anti-inflammatory and anti-infection effect of hybrid hydrogels [59]. TGF-β plays an important role in stimulating cell proliferation and ECM synthesis, while VEGF contributes to the regulation of angiogenesis, re-epithelization, and collagen synthesis during the healing process [43]. We observed that both the expression of TGF-β and VEGF in the hydrogel-treated groups were higher than that in the PBS group, and the CPGH group showed an advantageous effect over the GH group on day 7 after treatment (Fig. 8C, D). This indicated that the hybrid hydrogels could facilitate the transformation of the wound from the inflammatory stage to the proliferative phase by down-regulating the expression of IL-6 and TNF-α, and up-regulating the expression of TGF-β and VEGF synergistically. Additionally, immunohistochemical staining of CD31 on day 14 was conducted to assess the angiogenesis of the regenerated tissue [56]. The density of formed blood vessels was significantly higher in the CPGH group, consistent with the expression of VEGF (Fig. 8E, F). With the incorporation of C60@PDA nanocomposites, hybrid hydrogels could improve vascularization for better nutrient transportation in the proliferation and remodeling phases, which might arise from the favorable anti-oxidative property [55]. Taken together, the designed hybrid hydrogel could regulate excessive ROS, modulate inflammatory responses, and improve angiogenesis, thereby achieving desired wound healing effects.

## Conclusion

In summary, we fabricated a novel fullerene-based hybrid hydrogel with in situ forming property and excellent ROS-scavenging capacity for wound dressing application. PDA coating strategy increased the dispersity of C60, and then C60@PDA was incorporated into the GelMA to develop hybrid hydrogels. In vitro studies demonstrated that the hybrid hydrogel displayed a porous structure and swelled rapidly to absorb water, and they also presented excellent antioxidant capacity, biocompatibility, and antibacterial activities. In a mouse full-thickness wound defect model, we found that the hybrid hydrogels significantly accelerated wound closure and promoted skin regeneration. Histological staining studies revealed that hybrid hydrogels achieved better healing effects by alleviating oxidative stress and inflammatory responses, as well as promoting re-epithelialization, collagen deposition and neovascularization. Overall, hybrid hydrogels based on fullerene and mussel chemistry show great potential as a bioactive wound dressing for cutaneous wound repair.

## Supplementary Information

**Additional file 1: Figure S1.** Thermogravimetric analysis (TGA) curves of C60 and C60@PDA. **Figure S2.** DPPH radical scavenging percentage of C60@PDA with different concentrations, and the representative photographs inset. **Figure S3.** Zeta potential of C60 and C60@PDA. **Figure S4.** Quantitative analysis of DCF relative fluorescence intensity (n = 4). **Figure S5.** H&E staining images of main organs, including heart, liver, spleen, lung and kidney on day 7 post-treatment. Mice without any treatment were defined as the control group. **Figure S6.** H&E staining images of main organs on day 14 post-treatment. Mice without any treatment were defined as the control group. **Figure S7.** H&E staining images of the regenerated skin tissue on day 21 post-treatment. **Figure S8.** Masson’s trichrome staining images of the regenerated skin tissue on day 7 post-treatment.

## Data Availability

The data in this work are available in the manuscript or Additional file or available from the corresponding author upon reasonable request.

## Abbreviations

ROS: Reactive oxygen species
GelMA: Gelatin methacryloyl
PDA: Polydopamine
ECM: Extracellular matrix
MA: Methacrylic anhydride
LAP: Lithium phenyl-2,4,6-trimethylbenzoylphosphinate
FTIR: Fourier transform infrared spectrometer
DPPH: 1,1-Diphenyl-2-picrylhydrazyl
DCFH-DA: 2′,7′-Dichlorodihydrofluorescein diacetate
DPBS: Dulbecco’s phosphate buffered saline
α-MEM: Minimum Essential Medium α
FBS: Fetal bovine serum
CCK-8: Cell counting kit 8
SEM: Scanning electron microscope
TEM: Transmission electron microscope
