# Supplementary material for: In situ forming ROS-scavenging hybrid hydrogel loaded with polydopamine-modified fullerene nanocomposites for promoting skin wound healing
Source: J Nanobiotechnology. 2023 Apr 13;21:129. doi: 10.1186/s12951-023-01879-2 (PMC10099971; doi:10.1186/s12951-023-01879-2)
Supplement: Supplementary file 1 — Additional file 1: Figure S1. Thermogravimetric analysis (TGA) curves of C60 and C60@PDA. Figure S2. DPPH radical scavenging percentage of C60@PDA with different concentrations, and the representative photographs inset. Figure S3. Zeta potential of C60 and C60@PDA. Figure S4. Quantitative analysis of DCF relative fluorescence intensity (n = 4). Figure S5. H&E staining images of main organs, including heart, liver, spleen, lung and kidney on day 7 post-treatment. Mice without any treatment were defined as the control group. Figure S6. H&E staining images of main organs on day 14 post-treatment. Mice without any treatment were defined as the control group. Figure S7. H&E staining images of the regenerated skin tissue on day 21 post-treatment. Figure S8. Masson’s trichrome staining images of the regenerated skin tissue on day 7 post-treatment. [file 12951_2023_1879_MOESM1_ESM.docx]

**Additional file 1**

In situ forming ROS-scavenging hybrid hydrogel loaded with polydopamine-modified fullerene nanocomposites for promoting skin wound healing

Xuan Chen^1^, Yihui Zhang^1^, Wei Yu^1^, Wenkai Zhang^1^, Haozheng Tang^2^, Wei-En Yuan^1^*

^1^Engineering Research Center of Cell & Therapeutic Antibody, Ministry of Education, School of Pharmacy

* Corresponding Author Wei-En Yuan, E-mail: yuanweien@sjtu.edu.cn

^2^Department of Bone and Joint Surgery, Department of Orthopedics, Renji Hospital, School of Medicine, Shanghai Jiao Tong University, 145 Shandong Middle Road, Shanghai 200001, China

**Table of contents**

**Fig. S1.** Thermogravimetric analysis (TGA) curves of C60 and C60@PDA.

**Fig. S2.** DPPH radical scavenging percentage of C60@PDA with different concentrations, and the representative photographs inset.

**Fig. S3.** Zeta potential of C60 and C60@PDA.

**Fig. S4.** Quantitative analysis of DCF relative fluorescence intensity (n = 4).

**Fig. S5.** H&E staining images of main organs, including heart, liver, spleen, lung and kidney on day 7 post-treatment. Mice without any treatment were defined as the control group.

**Fig. S6.** H&E staining images of main organs on day 14 post-treatment. Mice without any treatment were defined as the control group.

**Fig. S7.** H&E staining images of the regenerated skin tissue on day 21 post-treatment.

**Fig. S8.** Masson’s trichrome staining images of the regenerated skin tissue on day 7 post-treatment.


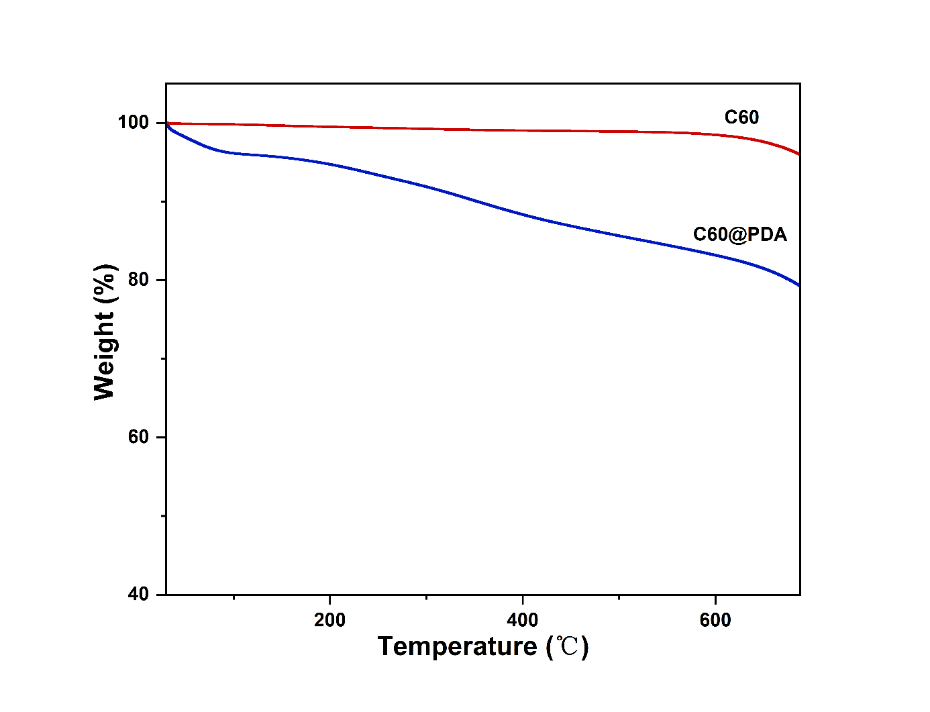


Fig. S1. Thermogravimetric analysis (TGA) curves of C60 and C60@PDA.


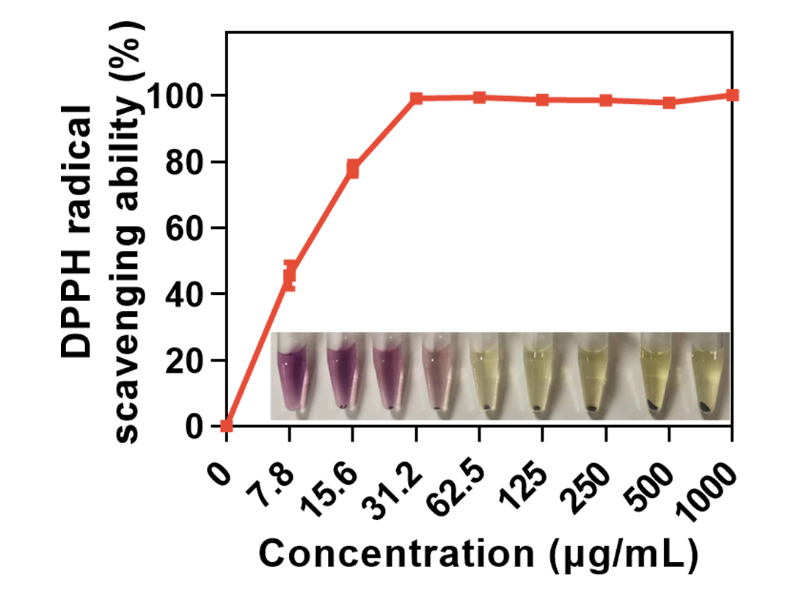


Fig. S2. DPPH radical scavenging percentage of C60@PDA with different concentrations, and the representative photographs inset.


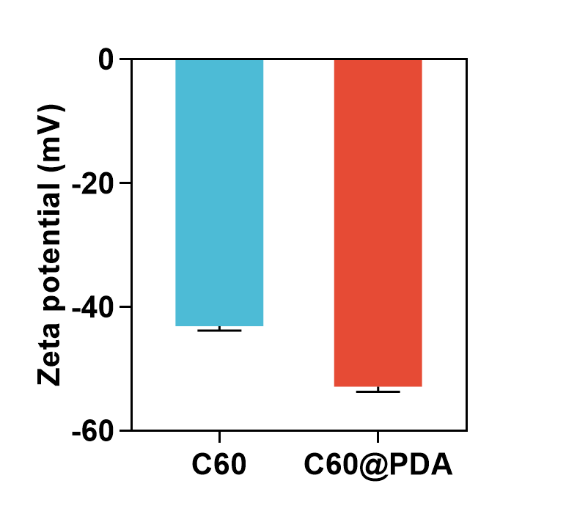


Fig. S3. Zeta potential of C60 and C60@PDA.


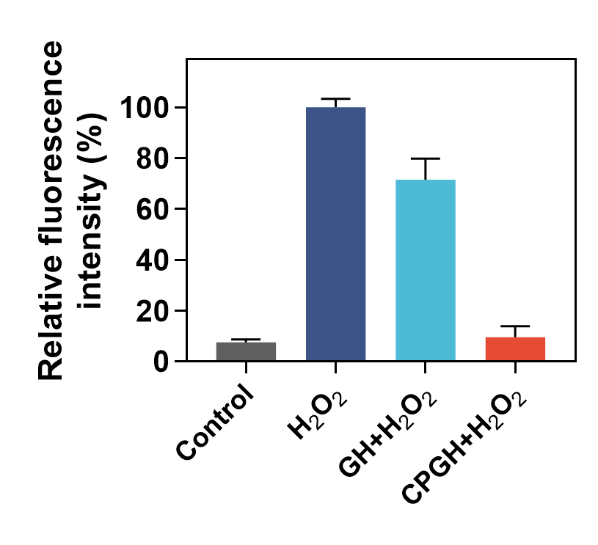


Fig. S4. Quantitative analysis of DCF relative fluorescence intensity (n = 4).


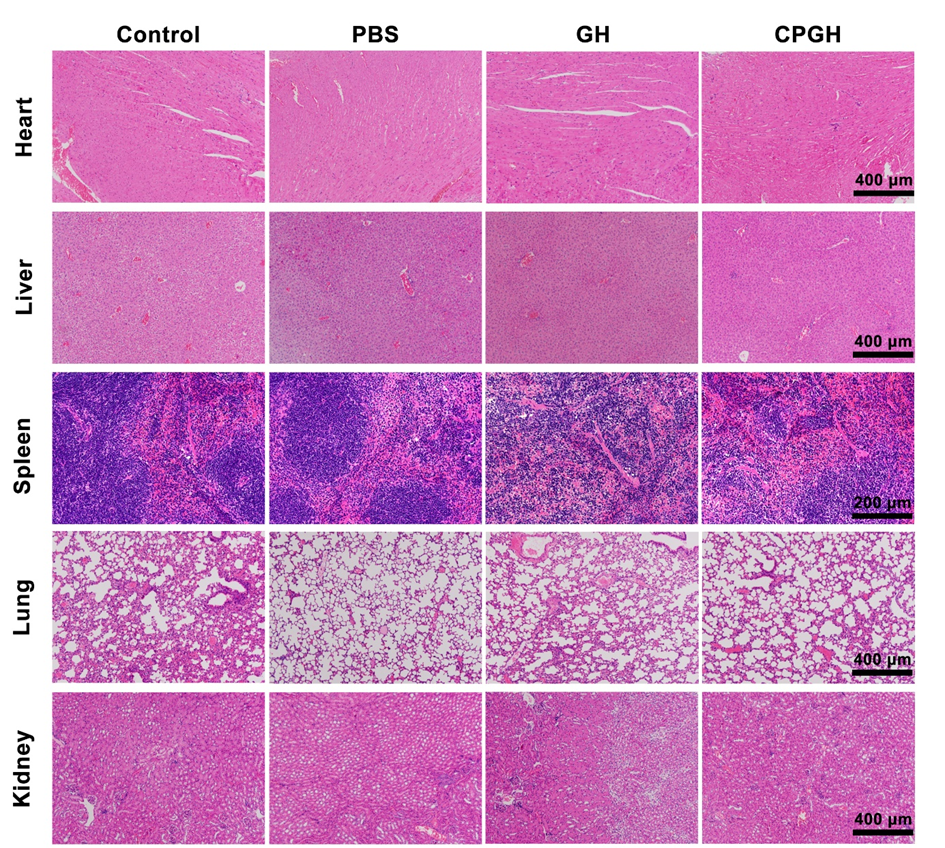


Fig. S5. H&E staining images of main organs, including heart, liver, spleen, lung and kidney on day 7 post-treatment. Mice without any treatment were defined as the control group.


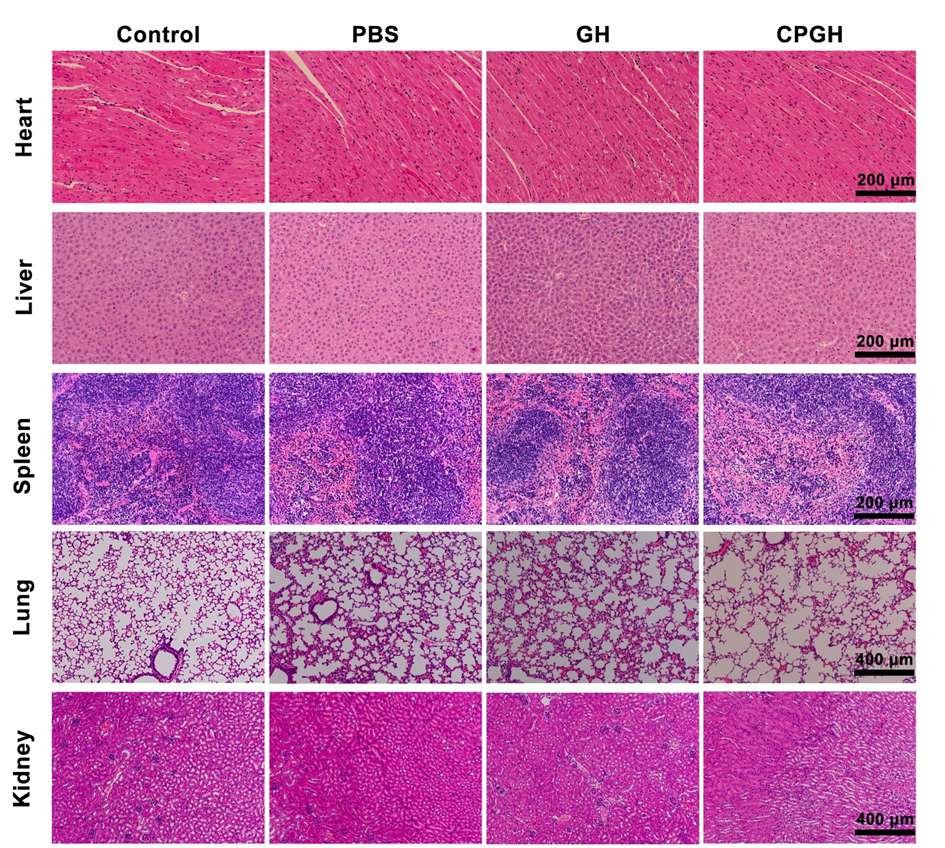


Fig. S6. H&E staining images of main organs on day 14 post-treatment. Mice without any treatment were defined as the control group.


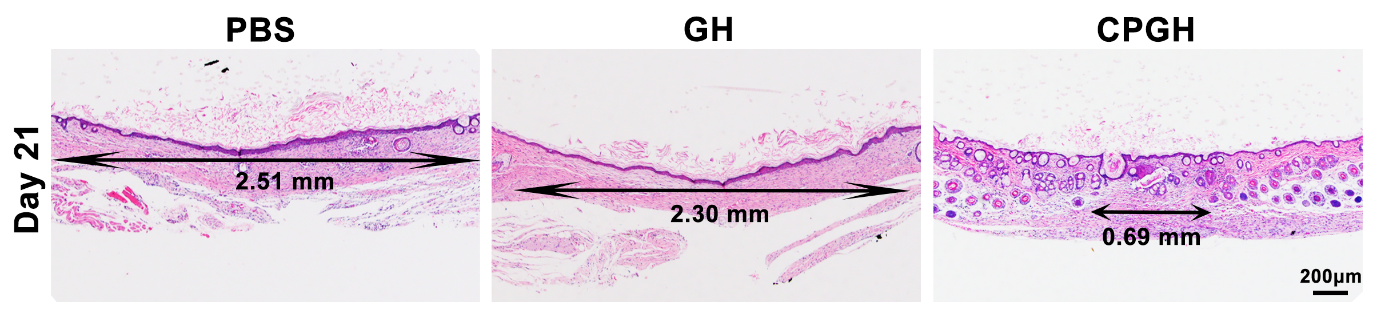


Fig. S7. H&E staining images of the regenerated skin tissue on day 21 post-treatment.


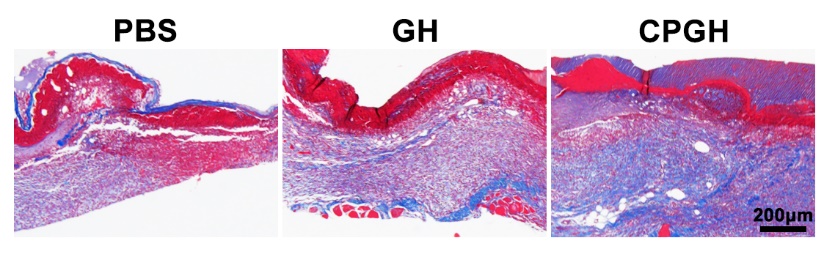


Fig. S8. Masson’s trichrome staining images of the regenerated skin tissue on day 7 post-treatment.
